# Elevated Neuronal Excitability Due to Modulation of the Voltage-Gated Sodium Channel Nav1.6 by Aβ_1−42_

**DOI:** 10.3389/fnins.2016.00094

**Published:** 2016-03-09

**Authors:** Xi Wang, Xiao-Gang Zhang, Ting-Ting Zhou, Na Li, Chun-Yan Jang, Zhi-Cheng Xiao, Quan-Hong Ma, Shao Li

**Affiliations:** ^1^Department of Physiology, Dalian Medical UniversityDalian, China; ^2^Department of Neurology, the First Affiliated Hospital of Dalian Medical UniversityDalian, China; ^3^The Key Laboratory of Stem Cell and Regenerative Medicine, Kunming Medical College, Institute of Molecular and Clinical MedicineKunming, China; ^4^Jiangsu Key Laboratory of Translational Research and Therapy for Neuro-Psycho-Diseases, Institute of Neuroscience, Second Affiliated Hospital, Soochow UniversitySuzhou, China

**Keywords:** Alzheimer's disease, beta-amyloid peptide, excitability, voltage-gated sodium channel, neurodegeneration

## Abstract

Aberrant increases in neuronal network excitability may contribute to the cognitive deficits in Alzheimer's disease (AD). However, the mechanisms underlying hyperexcitability are not fully understood. Such overexcitation of neuronal networks has been detected in the brains of APP/PS1 mice. In the present study, using current-clamp recording techniques, we observed that 12 days *in vitro* (DIV) primary cultured pyramidal neurons from P0 APP/PS1 mice exhibited a more prominent action potential burst and a lower threshold than WT littermates. Moreover, after treatment with Aβ_1−42_ peptide, 12 DIV primary cultured neurons showed similar changes, to a greater degree than in controls. Voltage-clamp recordings revealed that the voltage-dependent sodium current density of neurons incubated with Aβ_1−42_ was significantly increased, without change in the voltage-dependent sodium channel kinetic characteristics. Immunohistochemistry and western blot results showed that, after treatment with Aβ_1−42_, expressions of Nav and Nav1.6 subtype increased in cultured neurons or APP/PS1 brains compared to control groups. The intrinsic neuronal hyperexcitability of APP/PS1 mice might thus be due to an increased expression of voltage-dependent sodium channels induced by Aβ_1−42_. These results may illuminate the mechanism of aberrant neuronal networks in AD.

## Introduction

Alzheimer's disease (AD) is the most frequent neurodegenerative disease and a common cause of dementia in elderly individuals. Various evidence suggests, that β-amyloid (Aβ) peptides play a causal role in AD's pathogenesis, but the underlying mechanisms remain unclear (Palop and Mucke, 2010). In prodromal AD patients, functional MRI has revealed increased activities in neural networks, rather than loss of activity (Putcha et al., 2011). Other evidence supports the view, that Aβ-induced excitotoxicity could be critically involved in the pathogenesis of AD (Ong et al., 2013). In Aβ-induced excitotoxicity, high levels of glutamate overexcite neurons and cause cell death (Choi, 1988; Olney et al., 1997). Aβ also enhances the sensitivity of neuron to glutamate, which increases the activity of neuronal networks, resulting in excitatory potentials and Ca^2+^ influx (Brorson et al., 1995). However, these changes cannot explain patients with early AD frequently alternating between sober and confused, because synaptic disruption, or regeneration cannot repeatedly occur within such short time periods. This phenomenon is more likely due to the abnormal neural network excitability.

Voltage-gated sodium channels (Nav) play an essential role in excitable cells. They are necessary components required to generate and propagate action potentials (Goldin et al., 2000; Yu and Catterall, 2003; Catterall et al., 2005). The 260 kDa α subunit is the main component of the voltage-gated sodium channel. Nine α subtypes, named Nav1.1-Nav1.9, are expressed in excitable cells (Goldin et al., 2000; Ragsdale, 2008). Among them, the Nav1.1, Nav1.2, and Nav1.6 subtypes are expressed in the adult brain and regulate voltage-dependent sodium currents across the plasma membrane. Nav1.1 is primarily localized in the neuronal somata of GABAergic neurons (Yu et al., 2006; Ogiwara et al., 2007). Nav1.2 shows preferentially high expression in unmyelinated fibers (Ragsdale, 2008). The Nav1.6 subtype, encoded by the gene SCN8A, is conspicuously expressed at the nodes of Ranvier and axon initial segments (Trimmer and Rhodes, 2004). Unique features of Nav1.6 include its contribution to the persistent current, resurgent current, and repetitive neuronal firing.

Our previous results showed that Nav1.6 interacts with amyloid precursor protein (APP), which undergoes abnormal proteolytic processing to generate Aβ (Xu et al., 2014). APP also increases the surface expression of sodium channels through a G_o_ protein-coupled JNK pathway (Liu et al., 2015). Due to this effect of APP, we hypothesized that the upregulatory effect of Aβ on neuronal excitability might be partially based on modulating the expression of sodium channels. As we expected, in cultured cortical neurons, Aβ_1−42_ increased the expression of sodium channels, particularly the Nav1.6 subtype. The increased voltage-dependent sodium current could decrease action potential threshold and increase the probability of action potential generation in response to synaptic excitation, thus increasing the excitability of the neuron.

## Materials and methods

### Animals

Newborn C57BL/6J mice were obtained from the Animal Center of Dalian Medical University. APP/PS1 transgenic mice were purchased from Jackson Laboratory (stock number 004462) and were maintained on a C57BL/6J background by crossing heterozygous transgenic mice with C57BL/6J breeders. All experiments were conducted in accordance with the National Institutes of Health Guide for the Care and Use of Laboratory Animals. All efforts were made to minimize the number of animals used and their suffering.

### Preparation of Aβ_1−42_

Lyophilized Aβ_1−42_ peptide (SIGMA, A9810) and reverse peptide Aβ_42−1_ (SIGMA, SCP0048) were diluted to 1 mg/ml using sterile PBS and incubated at 37°C, 220 rpm for 48 h allowed to aggregate as described (Jones et al., 2013). For concrete forms of Aβ_1−42_ peptide, see Supplementary Figure S1. In a further set of experiments primary cultured neurons were incubated with Aβ_1−42_ (5.0 μM) or reverse peptide Aβ_42−1_ (5.0 μM) for 24 h at 37°C.

### Cell culture

P0 (post-natal day 0–1) C57BL/6J mice or APP/PS1 mice and their littermates were sacrificed by CO_2_ inhalation and the cortices were rapidly dissected under sterile conditions in cold PBS. The tissue was digested with 0.125% trypsin-EDTA at 37°C for 30 min. The trypsin solution was replaced with 2 ml 10% DMEM (DMEM with 10% FBS, 1% L-glutamine, and 1% penicillin/streptomycin solution). The digested tissue was gently triturated by suction using a glass pipette flamed on the tip to avoid cellular damage. The cell suspension was filtered through a 74 μm screen mesh, plated on poly-L-lysine-coated coverslips or 6/12-well-plates and incubated in a 37°C, 5% CO_2_ incubator. After 2 h, the medium was changed to Neurobasal (Gibco, 21103–049) supplemented with 2% B27, 1% L-glutamine, and 1% penicillin/streptomycin. In accordance with the routine culture, the medium was changed every 2 days until use.

### MTT assay

Cell viability of neurons was determined by the MTT assay. Neurons were plated in 96 well-plates. Aβ_42−1_ and Aβ_1−42_ peptides were added to wells for 24 h. After cell treatments the medium was removed and the cells were incubated with red free medium and MTT solution (0.5 mg/ml) for 4 h at 37°C. Finally, the medium was removed and formazan particles were dissolved in DMSO. Cell viability, defined as the relative amount of MTT reduction was determined by spectrophotometry at 570 nm.

### Electrophysiological recordings

Electrophysiological measurements were performed on pyramidal cells. Action potentials or sodium currents were recorded at room temperature using whole-cell patch-clampings. The extracellular solution contained the following (in mM): NaCl 150, KCl 5, MgCl_2_ 1.1, CaCl_2_ 2.6, HEPES 10, glucose 10, pH adjusted to 7.4 with NaOH. Patch pipettes were made from borosilicate glass capillaries (1.5 mm outer diameter, 0.8 mm inner diameter) using a micropipette puller (Narishige, PP 830, Japan). Pipette resistance ranged from 3 to 5 MΩ. Stimulation and data acquisition were performed using the EPC-10 patch-clamp amplifier and Pulse program (HEKA Electronik, Germany). Membrane currents were filtered at 2 kHz and digitized at 10 kHz.

Action potentials recordings were made using the current-clamp mode. The intracellular solution contained the following (in mM): KCl 65, KOH 5.0, KF 80, HEPES 10, EGTA 10, Na_2_ATP 2, pH adjusted to 7.2 with KOH. Cells were held at −70 mV, then peak amplitude (80 pA, 10 ms), threshold (80 pA, 10 ms), and action potential firing (200 pA, 500 ms) were recorded and measured.

Sodium currents were recorded using the voltage-clamp mode. The intracellular solution contained the following (in mM): CsCl 140, MgCl_2_ 2, Na_2_ATP 2, EGTA 10, HEPES 20, pH adjusted to 7.2 with Tris-HCl. Cells were held at −70 mV and stepped to a range of potentials (−60 to +60 mV in 10 mV increments) for 12 ms each. Peak inward currents (I) were plotted as a function of depolarizing potential to generate I–V curves. Activation curves were obtained by converting current (I) to conductance (G) at each voltage (V) using the equation G = I/(V − V_rev_), where V_rev_ is the reversal potential, which was determined for each cell individually. Activation curves were then fit with the Boltzmann function in the form of G/G_max_ = 1/{1 + exp [(V_1∕2_ − V)/κ]}, where G_max_ is the maximal sodium conductance, V_1∕2_ is the half-maximal activation potential, V is the test potential, and κ is the slope factor. Steady-state fast inactivation was achieved with a series of 500 ms prepulses (−120 to −10 mV in 10 mV increments), and the remaining available channels were activated by a 12 ms test pulse to 0 mV. Peak inward currents obtained from steady-state fast-inactivation protocols were normalized to the maximal peak current (I_max_) and fit with Boltzmann functions: I/I_max_ = 1/{1 + exp [(V − V_1∕2_)/κ]}, where V represents the inactivating prepulse potential, and V_1∕2_ represents the mid-point of inactivation curve.

### Data analysis

Data were analyzed using Pulsefit 8.6 and Origin 7.5, and presented as means ± SEM. The Kruskal–Wallis non-parametric test was used to analyze current density data. One-way ANOVA was used to assess the statistical significance of changes in characteristics of channel activation and inactivation. Statistical comparisons were performed by Student's *t*-test.

### Western blot

All cells were lysed in RIPA buffer (with 1% PMSF), and total protein concentrations were determined with a BCA Protein Assay Kit (TransGen). Total protein (10–20 mg) for each sample was loaded into precast 8% SDS-PAGE gels and run with running buffer. Gels were transferred onto PVDF membranes (Millipore). Antigen-specific primary antibodies (Pan sodium channel, Chemicon-AB5210; Nav1.6, Chemicon-AB5580 and Abcam-ab65166; β-tubulin, Abcam-ab6046; β-actin, Abcam-ab6276) were incubated overnight at 4°C and detected with species-specific horseradish-peroxidase-labeled secondary antibodies. An ECL Western Blotting Detection kit (TIANGEN) was used to obtain a chemiluminescence signal, which was detected using Gel Imaging System (Bio-Rad). Band quantification was performed using a Gel-Pro software. Bands of interest were normalized to actin- or tubulin- for a loading control.

### RT-PCR

Total RNA of cultured neurons was prepared by using the Trizol Reagent (Invitrogen, USA). The Superscript TM-III kit (Invitrogen, USA) was used for reverse-transcribed with oligo dT and 2.5 mg total RNA. Primer sequences were as follows: NM_019266 (SCN8A), and NM_031144 (β-actin). SCN8A forward 5′-CTG GAG AAT GGA GGC ACA CAC-3′, reverse 5′-ACG CTG CTG CTT CTC CTT GTC-3′; and β-actin forward 5′-CGT TGA CAT CCG TAA AGA CCT-3′, reverse 5′-TCA GGA GGA GCA ATG ATC TTG-3′. The resulting cDNA PCR amplification was performed by using the following protocol: 95°C for 10 s followed by 50 cycles of 95°C for 5 s and 60°C for 31 s, and verified by 2.0% agarose gel electrophoresis. Images were captured by using Gel Imaging System (Bio-Rad). The amplicon size of each gene was108 and 144 bp, respectively.

### Immunofluorescence staining and immunohistochemistry

Cells were fixed with 4% paraformaldehyde for 20 min, permeabilized with 0.1% Triton-x-100 for 5 min, and incubated with 5% BSA for 60 min at room temperature to block non-specific binding. Without washing, the diluted primary antibodies (MAP2, Abcam-ab32454; Nav1.6, Abcam-ab65166) were added and incubated at 4°C overnight. After three washes with PBS, cells were incubated with the corresponding secondary antibodies at room temperature for 1 h. For immunohistochemistry, cells were stained with DAB kit (Vector Laboratories) according to the instructions of the manufacturer for peroxidase labeling. Images were acquired from a fluorescence microscopy (Leica Microsystems DM400B, Germany).

### Image analysis and quantification

Quantitative analysis of mean fluorescence intensities (MFIs) of immunoreactive neurons was performed using Image J software (National Institutes of Health). MFI per square micrometer was calculated by dividing the MFI units by the area of outlined regions.

## Results

### Elevated excitability in APP/PS1 mice

**Figure 1:**
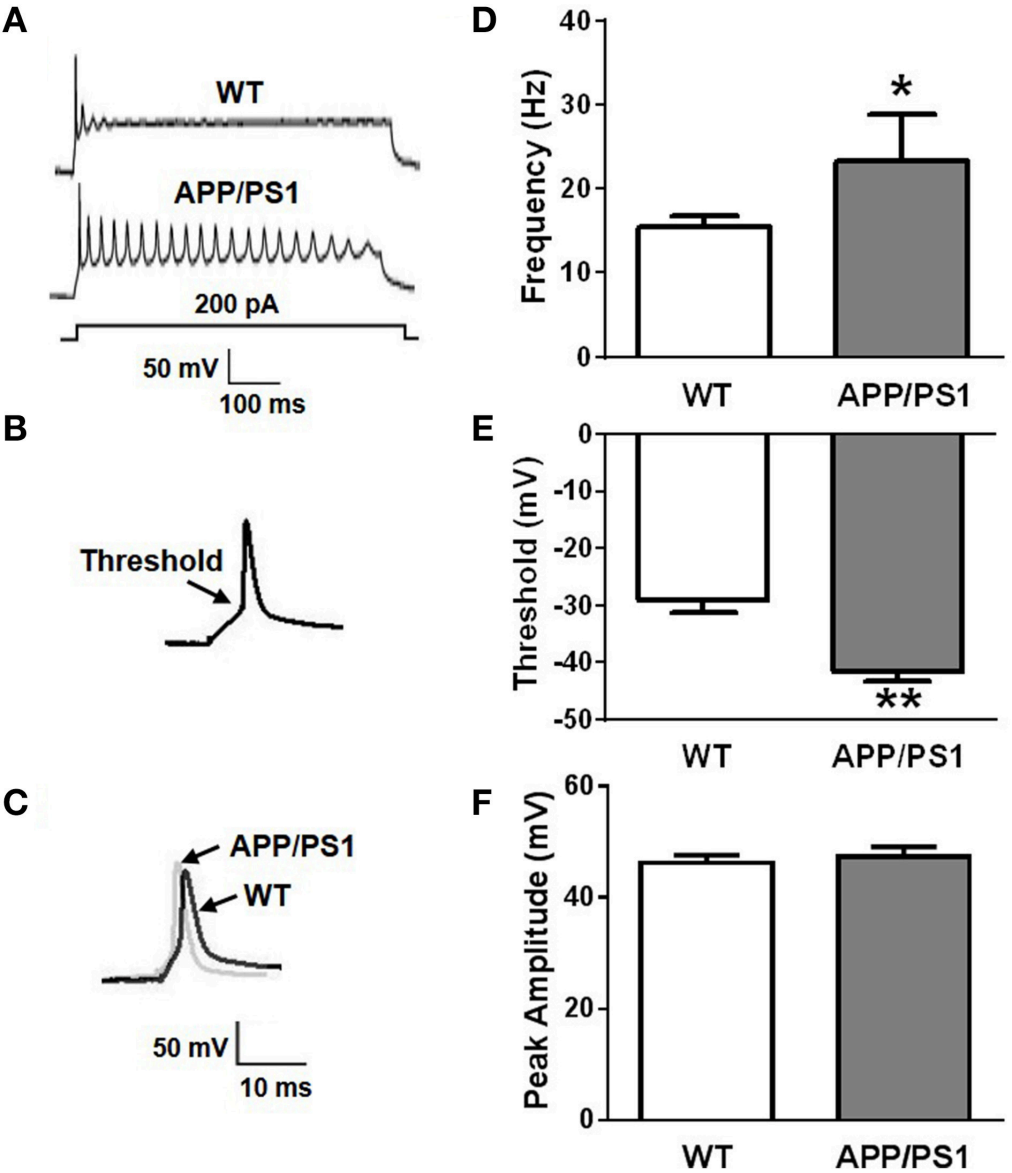
**Increased neuronal excitability in APP/PS1 mice. (A)** Representative recording traces of action potential repetitive firing. **(B)** The schematic of threshold. **(C)** Representative recording traces of action potential peak amplitude. **(D)** The mean number of APs elicited by 200 pA depolarizing current. Neurons from APP/PS1 mice (*n* = 10) fired significantly more APs than WT (*n* = 12). **(E)** AP threshold in APP/PS1 mice cultured pyramidal neurons was significantly lower than WT mice (*n* = 9 per group). **(F)** The peak amplitude of APs in WT and APP/PS1 (*n* = 9 per group) mice cultured pyramidal neurons didn't show significantly difference. Mean ± SEM was displayed. ^*^Presents p < 0.05; ^**^presents *p* < 0.01.

To investigate change in intrinsic excitability in APP/PS1 mice, whole-cell patch-clamp recordings were performed on 12 DIV (days *in vitro*) primary cultured neurons obtained from P0 APP/PS1 mice and littermate wide type mice in current-clamp mode. Frequency, threshold, and peak amplitude of action potentials (AP) were examined by different protocols. AP firing frequencies (f) elicited by increasing depolarizing currents (200 pA, 500 ms) were significantly increased in neurons from APP/PS1 mice compared to WT mice (Figures 1A,D; f_WT_ = 15.375 ± 3.428 Hz, *n* = 12; f_APP∕PS1_ = 23.25 ± 7.264 Hz, *n* = 10; *p* = 0.0128). We used a depolarizing current (80 pA, 10 ms) to induce a single firing of an AP. Threshold potential (V_threshold_) and amplitude of action potential (V_peak_) were recorded and compared with WT. We found that thresholds of neurons from APP/PS1 mice were significantly decreased compared to WT mice (Figures 1B,E; V_threshold, WT_ = −29.001 ± 2.304 mV, *n* = 9; V_threshold, APP∕PS1_ = −41.601 ±1.965 mV, *n* = 9; *p* = 0.0012), but the peak amplitude was not (Figures 1C,F; V_peak, WT_ = 46.159 ± 2.663 mV, *n* = 9; V_peak, APP∕PS1_ = 47.236 ± 3.849 mV, *n* = 9; *p* = 0.6475). These results suggested that the excitability of mature neurons obtained from APP/PS1 mice was increased.

### Aβ_1−42_ increases excitability in cultured cortical neurons from mice

**Figure 2:**
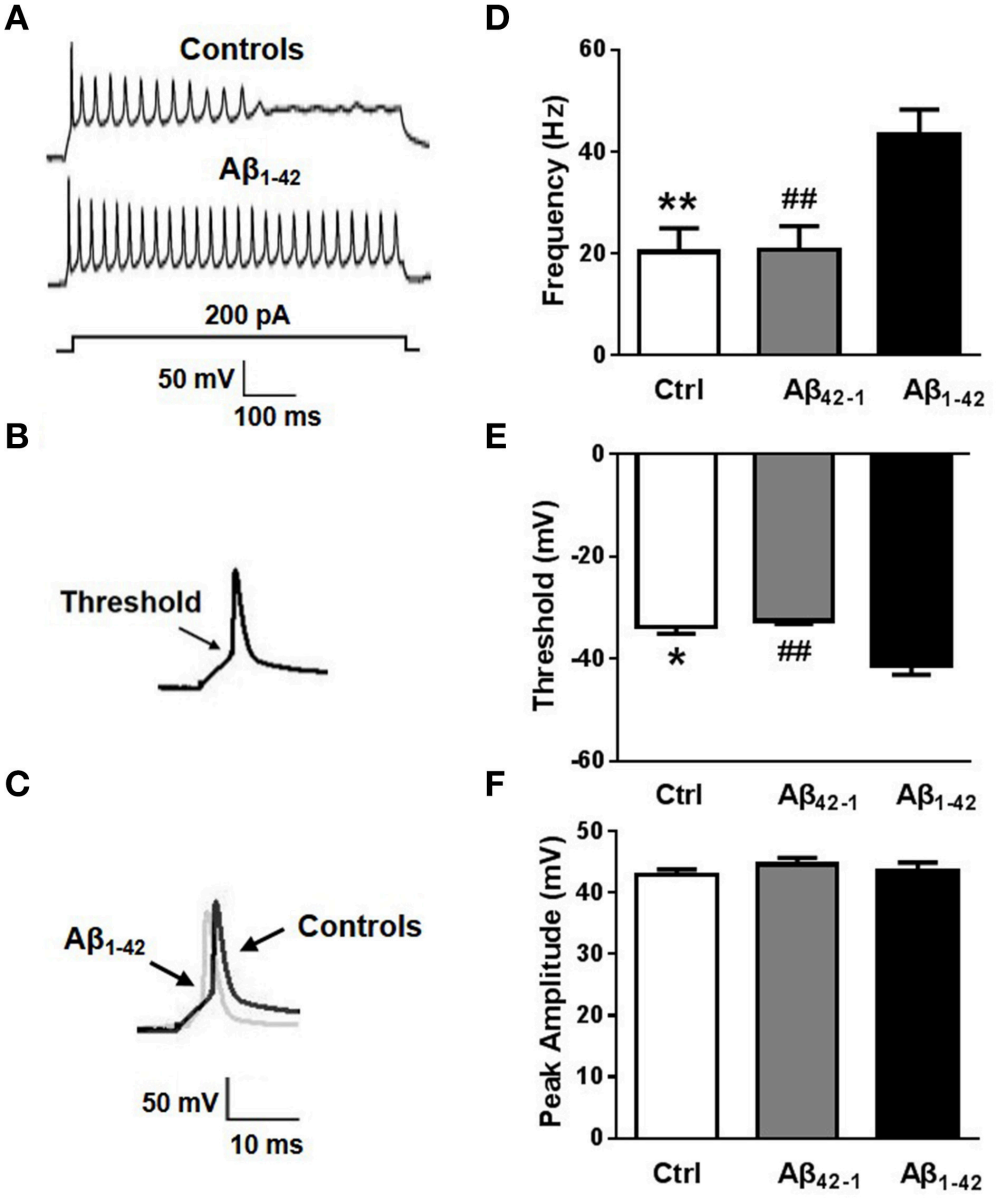
**Aβ affects neuronal excitability. (A)** Representative recording traces of action potential repetitive firing. **(B)** The schematic of threshold. **(C)** Representative recording traces of action potential peak amplitude. **(D)** The mean number of APs elicited by 200 pA depolarizing current. Neurons after Aβ_1−42_ treatment (*n* = 8) fired significantly more APs than controls (both negative control group and reverse peptide Aβ_42−1_ group, *n* = 8). **(E)** AP threshold in pyramidal neurons treated with Aβ_1−42_ (*n* = 7) was significantly lower than controls (*n* = 7). **(F)** The peak amplitude of APs in pyramidal neurons treated with Aβ_1−42_ (*n* = 7) didn't show significantly difference with controls (*n* = 7). Mean ± SEM was displayed. ^*^Presents *p* < 0.05 vs. control group; ^**^presents *p* < 0.01 vs. control group; ^*##*^presents *p* < 0.01 vs. Aβ_1−42_ group.

It is likely that diverse factors contribute to the pathogenesis of AD patients or mice (Blennow et al., 2006; Bertram and Tanzi, 2008; Mucke, 2009). Among them, Aβ stands out on the basis of overwhelming genetic evidence and strong experimental data (Farrer et al., 1997; Hardy and Selkoe, 2002; Tanzi and Bertram, 2005; Mahley and Huang, 2006). Accordingly, we attempted to investigate whether Aβ_1−42_ contributed to intrinsic excitability. Cell viability was firstly determined by MTT assay in primary cultured neurons treated with 5 μM Aβ_42−1_ or 5 μM Aβ_1−42_ for 24 h. We found that Aβ_1−42_ slightly induced loss of neuron viability (Supplementary Figure S2; *n* = 3, means three independent experiments; *p* = 0.047). As described previously, whole-cell patch-clamp recordings were performed in current-clamp mode on normal morphological primary neurons which were incubated with Aβ_1−42_ or Aβ_42−1_ for 24 h. Frequency, threshold, and peak amplitude of AP were examined. We found AP firing frequencies were significantly increased in neurons after treatment with Aβ_1−42_ peptide compared to controls (Figures 2A,D; f_control_ = 20.25 ± 4.742 Hz, *n* = 8, *p* = 0.0050; f_Aβ42−1_ = 20.75 ± 4.644 Hz, *n* = 8, *p* = 0.0054; f_Aβ1−42_ = 43.25 ± 5.028 Hz, *n* = 8). Aβ_1−42_ also significantly decreased the threshold (Figures 2B,E; V_threshold, control_ = −33.83 ± 1.207 mV, *n* = 7, *p* = 0.0126; V_threshold, Aβ42−1_ = −32.55 ± 0.6600 mV, *n* = 7, *p* = 0.0035; V_threshold, Aβ1−42_ = −41.37 ± 1.771 mV, *n* = 7). But it had no effect on the peak amplitude (Figures 2C,F; V_peak, control_ = 42.83 ± 0.9437 mV, *n* = 7, *p* = 0.6579; V_peak, Aβ42−1_ = 44.60 ± 1.051 mV, *n* = 7, *p* = 0.5633; V_peak, Aβ1−42_ = 43.58 ± 1.305 mV, *n* = 7). These results suggested that incubation in Aβ_1−42_ increases neuronal excitability *in vitro*.

### Increased neuronal excitability induced by Aβ_1−42_ due to an up-regulation of nav current

**Figure 3:**
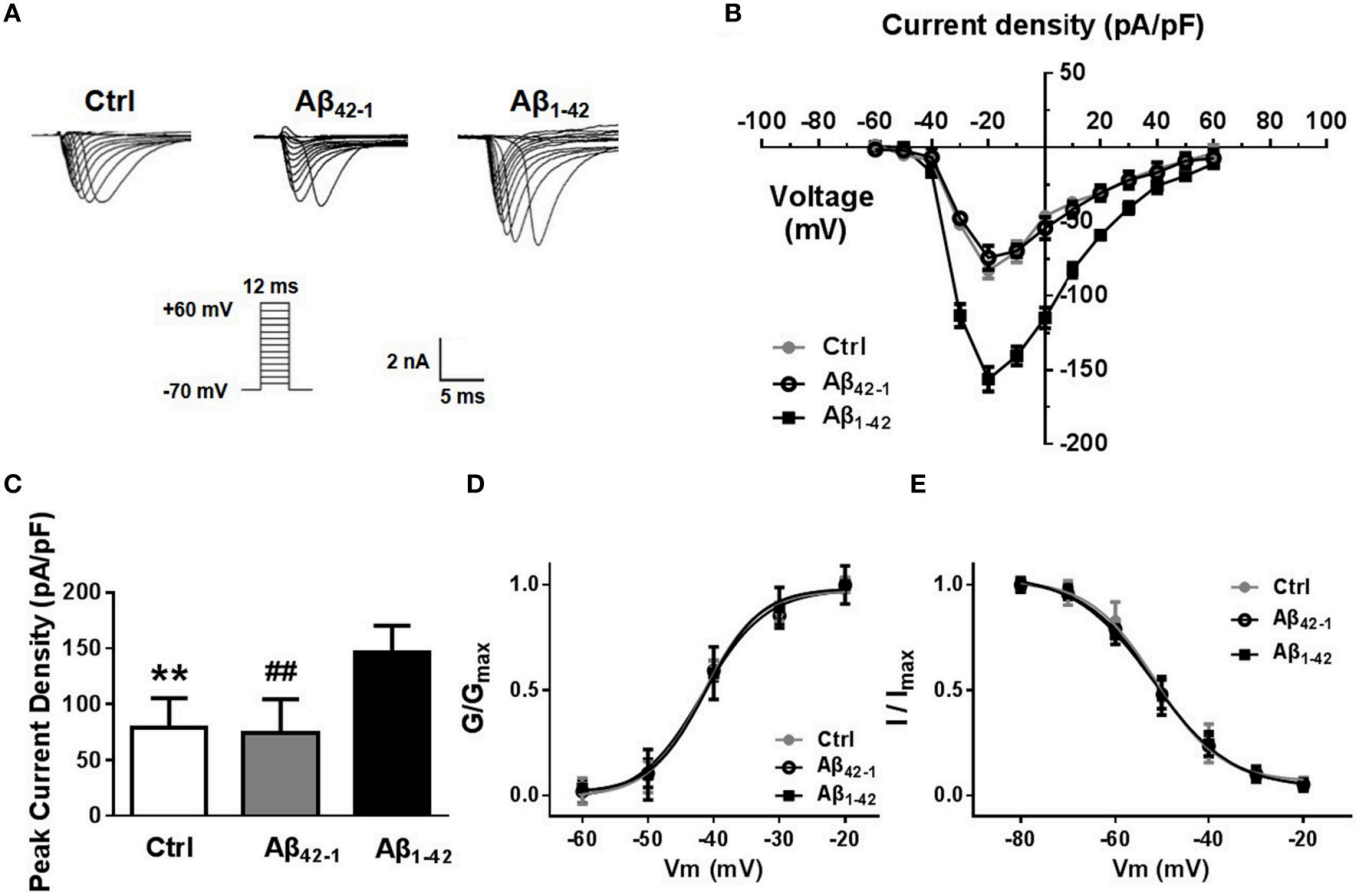
**Aβ_1−42_ up-regulates voltage-dependent sodium current. (A)** Representative traces of voltage gated sodium currents recorded in response to voltage steps from −60 to +60 mV. **(B)** Current density-voltage relationship, illustrates that Na^+^ current density recorded from Aβ_1−42_ treatment pyramidal neurons was significantly bigger than counterparts (negative control group and reverse peptide Aβ_42−−1_ group, *n* = 12). **(C)** Graph, data showed peak current density recorded from Aβ_1−42_ treatment neurons was significantly bigger than counterparts (*n* = 12). **(D)** Normalized steady-state activation curves for Na^+^ currents recorded from cultured pyramidal neurons after Aβ_1−42_ treatment and counterparts (*n* = 12). The activation curves were not significantly shift after Aβ_1−42_ treatment. **(E)** Normalized steady-state inactivation curves for Na^+^ currents recorded from these three groups, and the inactivation curve was not significantly shift after Aβ_1−42_ treatment either. Mean ± SEM was displayed. ^**^Presents *p* < 0.01 vs. Aβ_1−42_ group; ^*##*^presents *p* < 0.01 vs. Aβ_1−42_ group.

In mammalian neurons, dense clusters of voltage-gated sodium channels (Nav) at the axonal initial segment and nodes of Ranvier underlie action potential generation and fast propagation (Leterrier et al., 2011). In addition, non-inactivating persistent sodium currents support maintained depolarization during and between action potentials. Finally, the resurgent persistent sodium current is triggered upon repolarization and supports repetitive firing in some types of neurons (Raman et al., 1997). We therefore hypothesized that the increased excitability induced by Aβ_1−42_ could be due, at least in part, to an upregulation of Nav current. To test this hypothesis, we evaluated the magnitude of Nav currents in voltage-clamp mode after incubation with Aβ_1−42_ or reverse peptide for 24 h. Representative traces of the total inward current recorded in response to voltage steps from −60 to +60 mV are shown in Figure 3A. Current density curves (Figure 3B) and peak current densities (Figure 3C) were significantly increased after Aβ_1−42_ treatment compared to controls (Peak_control_ = 79.18 ± 11.60 pA/pF, *n* = 12, *p* = 0.0028; Peak_Aβ42−1_ = 74.14 ± 13.54 pA/pF, *n* = 12, *p* = 0.0031; Peak_Aβ1−42_ = 146.3 ± 10.70pA/pF, *n* = 12).

We next investigated the effect of Aβ_1−42_ on sodium channel kinetic characteristics (Figures 3D,E). We found that Aβ_1−42_ did not significantly shift the voltage-dependent sodium activation curve (Figure 3D). Single Boltzmann distribution fits showed a V_1∕2, *control*_ = −41.62 ± 1.462 mV, and a κ of 4.165 ± 1.548 mV (*n* = 12); a V_1∕2, Aβ42−1_ = −41.71 ± 1.768 mV and a κ of 4.795 ± 1.825 mV (*n* = 12); and a V_1∕2, Aβ1−42_ = −41.19 ± 1.052 mV and a κ of 4.155 ± 1.184 mV (*n* = 12). For steady-state inactivation curves (Figure 3E), the V_1∕2_ and κ of inactivation also did not change significantly (V_1∕2, control_ = −51.47 ± 1.040 mV and κ = 6.992 ± 1.017 mV, *n* = 12; V_1∕2, Aβ42−1_ = −51.90 ± 0.8201 mV and κ = 8.384 ± 0.8552 mV, *n* = 12; V_1∕2, Aβ1−42_ = −51.40 ± 0.4131 mV and κ = 7.723 ± 0.4178 mV, *n* = 12).

Overall, these results demonstrated that exposure of neurons to Aβ_1−42_ leads to an increased neuronal excitability, likely through a Nav current-mediated mechanism.

### Increased expression of nav in cultured neurons and APP/PS1 mice

**Figure 4:**
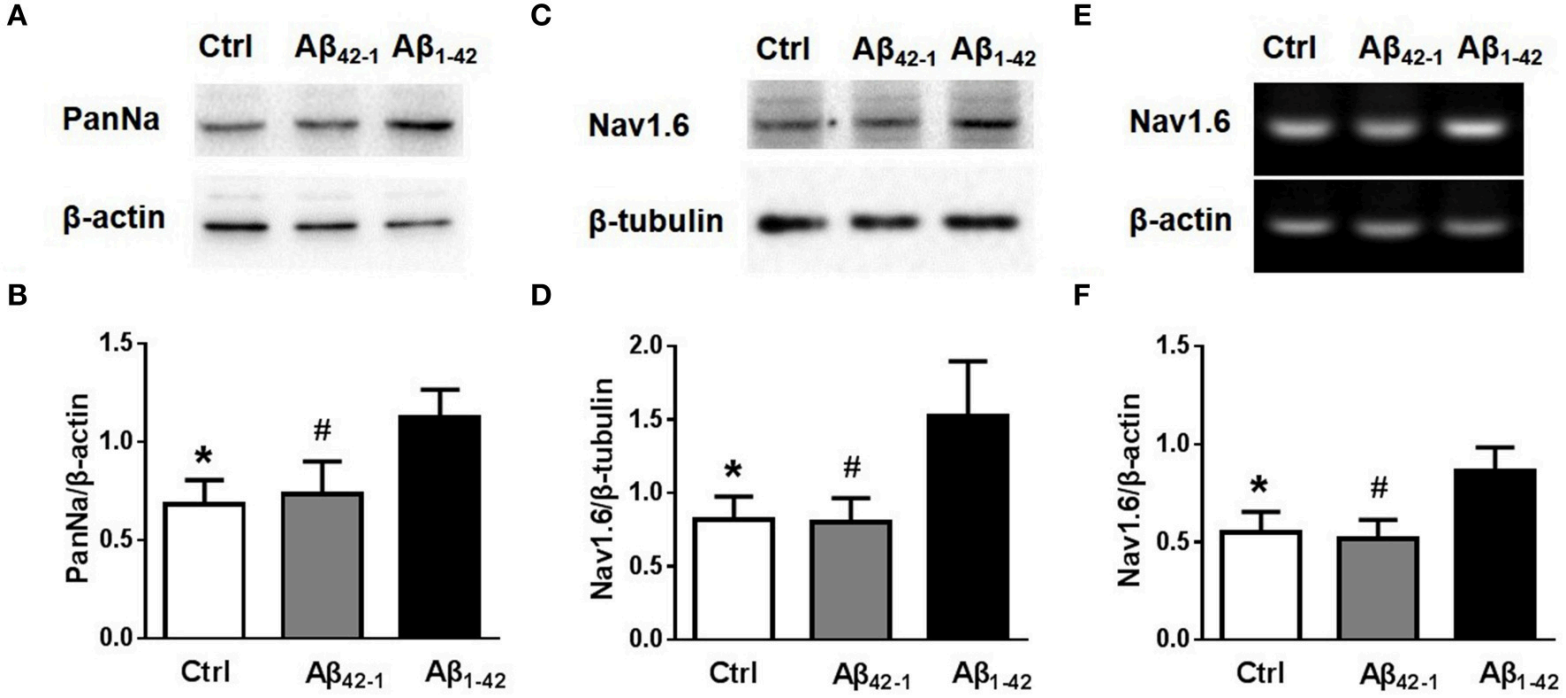
**Aβ_1−42_ increases the expression of sodium channels. (A)** Equal amounts of collected protein samples obtained from 12 DIV neurons after different treatments were analyzed by western blot to detect the protein expression level of sodium channel (*n* = 3 per group). **(B)** Quantification of protein levels of sodium channel. β-actin was used as an internal control (*n* = 3 per group). **(C)** Western blots of 12 DIV neurons after different treatments to detect the expression of Nav1.6 protein (*n* = 3 per group). **(D)** Quantification of protein levels of Nav1.6. β-tubulin was used as an internal control (*n* = 3 per group). **(E)** RT-PCR of Nav1.6 mRNA levels of 12 DIV neurons after different treatments (*n* = 3 per group). **(F)** Quantification of mRNA levels of Nav1.6. β-actin was used as an internal control (*n* = 3 per group). Mean ± SEM was displayed. ^*^Presents *p* < 0.05 vs. Aβ_1−42_ group; #presents *p* < 0.05 vs. Aβ_1−42_ group.

Although, the experiments described above revealed that Aβ_1−42_ increased neuronal excitability, perhaps dues to increasing Nav currents, it remained unclear how the Nav currents increased. There was no observable difference in sodium channel kinetic characteristics after Aβ_1−42_ incubation (Figures 3D,E). We then examined the expression of Nav in neurons after Aβ_1−42_ treatment. Using Western blot analysis of cultured cortical neurons, we found that incubation with Aβ_1−42_ significantly increased the expression of Nav [Figures 4A,B; *n* = 3; *p*_Ctrl vs. Aβ1−42_ = 0.0146; *p*_Aβ42−1 *vs*. *Aβ*1−42_ = 0.0358]. We also investigated the Nav1.6 subtype, which plays a major role in the transmission of subthreshold currents, namely the persistent and resurgent currents (Raman et al., 1997), and the electrophysiological properties of Nav1.6 make these channels especially suited for the sustained repetitive firing of neurons (Van Wart and Matthews, 2006). As shown in Figures 4C,D, Nav1.6 displayed a significant increase after Aβ_1−42_ treatment [*n* = 3; *p*_Ctrl vs. Aβ1−42_ = 0.0372; *p*_Aβ42−1 vs. Aβ1−42_ = 0.0354]. To further identify the increased expression of Nav1.6 after Aβ_1−42_ treatment, mRNA expression levels were detected too. Paralleled with western blot analysis, Nav1.6 mRNA obtained from cultured neurons treated with Aβ_1−42_ showed significantly increased than control groups [Figures 4E,F, *n* = 3; *p*_Ctrl vs. Aβ1−42_ = 0.0257; *p*_Aβ42−1 *vs*. *Aβ*1−42_ = 0.017].

**Figure 5:**
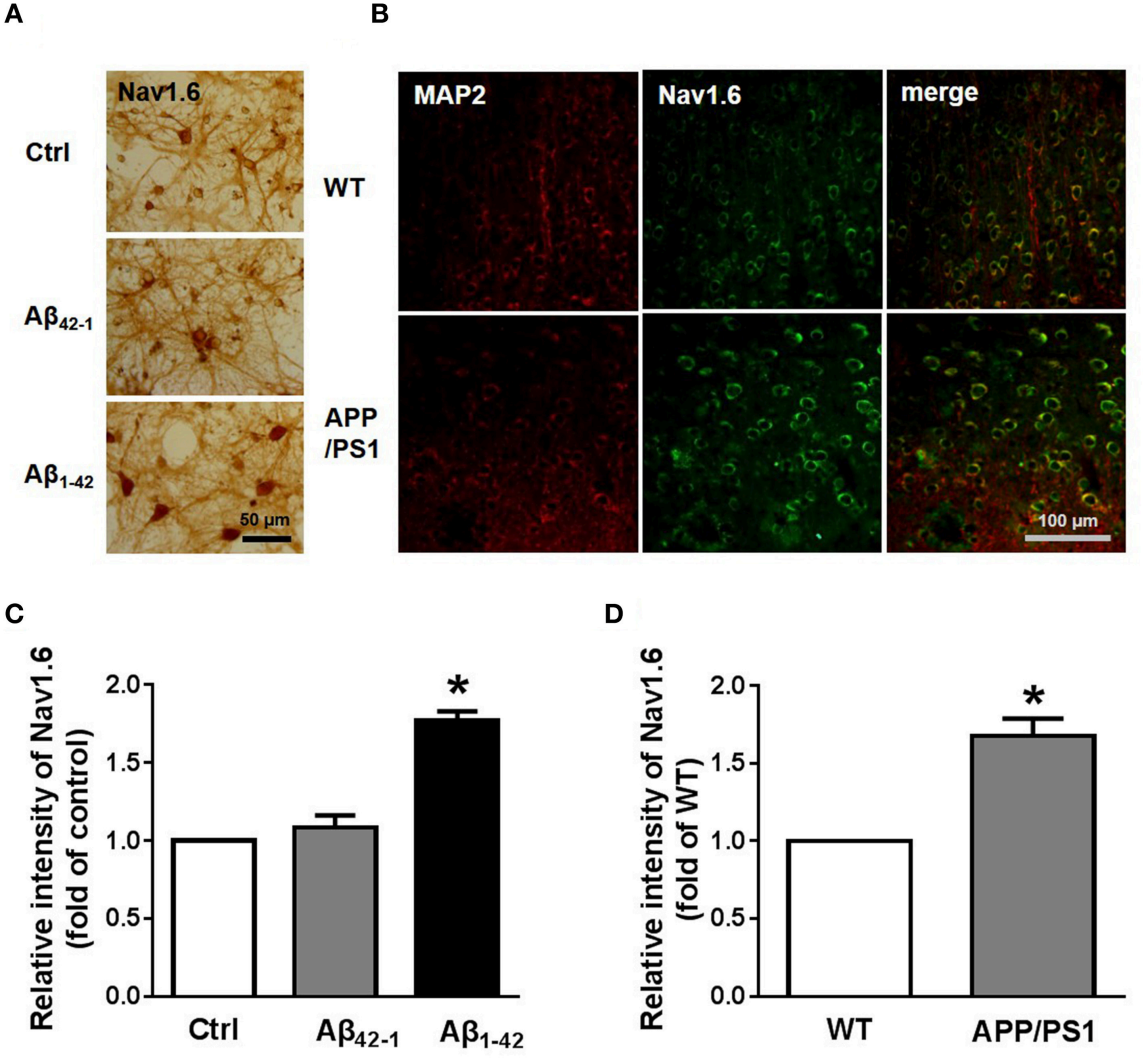
**Elevated expression of Nav1.6 in cultured neurons and APP/PS1 mice. (A)** Immunohistochemistry images of 12 DIV primary cortical culture neurons after 24 h Aβ_42−1_ or Aβ_1−42_ treatments stained with anti-Nav1.6 antibody. Scale bar = 50 μm (*n* = 3 per group). **(B)** Immunostaining images of temporal lobe cortex sections obtained from 9 months APP/PS1 mice and its littermates co-stained with anti-Nav1.6 antibody and anti-MAP2 antibody (*n* = 3 per group). **(C)** Quantification of Nav1.6 immunohistochemistry images by using intensity analysis (*n* = 3 per group). **(D)** Quantification of fluorescence intensity of Nav1.6 (*n* = 3 per group). Mean ± SEM was displayed. ^*^Presents *p* < 0.05 vs. control group.

Morphological methods were also used to test the expression of Nav1.6. The localization of Nav1.6 in neurons was revealed by immunohistochemistry (Figure 5A). Consistent with the result from western blotting, most neurons incubated with Aβ_1−42_ showed significantly deepening stain [Figures 5A,C; *n* = 3; *p*_Ctrl vs. Aβ1−42_ = 0.0162]. After obtaining these results, we wondered if the expression of Nav1.6 in APP/PS1 mouse brains would have changed. Coronal brain sections from 9 month old APP/PS1 mice and WT littermates were stained for MAP2, a maker of mature neurons, and Nav1.6 (Figure 5B; *n* = 3). We found, that APP/PS1 mice exhibited more Nav1.6 immunoreactivity than WT mice in cortex regions (Figure 5D; *n* = 3; *p* = 0.0286).

Taken together, our findings and previous studies suggest that Aβ_1−42_ increased the excitability of cultured cortical neurons, and this effect was mediated by overexpression of Nav, with the Nav1.6 subtype perhaps accounting for much of this increase.

## Discussion

In addition to cognitive deficits, AD patients have an increased incidence of epileptic seizures. This incidence is even higher in patients with early-onset AD who overexpress human APP, the proteolysis of which generates Aβ (Palop and Mucke, 2009). Hyperexcitability is also detected in the brains of various AD transgenic mice (Tamagnini et al., 2015), including the APP/PS1 mice used here. Such aberrant increases in network excitability and compensatory inhibitory mechanisms in the hippocampus may contribute to the cognitive deficits in AD (Palop et al., 2007; Sanchez et al., 2012; Verret et al., 2012). However, the mechanisms underlying the hyperexcitability detected in AD brains are not fully understood.

In the present study, we investigated the excitability in cultured pyramidal neurons of APP/PS1 mice using patch clamp techniques. We found that the excitability of 12 DIV cultured pyramidal neurons from APP/PS1 mice was significantly greater than in WT littermates. Additionally, AP firing frequencies increased and V_threshold_ decreased in neurons from APP/PS1 mice compared to WT mice. These results confirm previous studies that showed neuronal hyperexcitability in APP/PS1 mice by patch clamp methods. In recent years, many studies demonstrated that Aβ is associated with increased excitability of neurons *in vitro* and in animal models, leading to hypersynchronous network activity and higher risk for seizures (Minkeviciene et al., 2009; Busche et al., 2012; Born et al., 2014; Davis et al., 2014). In our current work, we observed the altered excitability of pyramidal neurons after treatment with Aβ. The higher firing frequency and lower threshold indicated an increased intrinsic excitability of pyramidal neurons.

Aβ-induced aberrant excitatory activity might occur through many different mechanisms. Previous studies have shown that Aβ can downregulate A-type K^+^ currents, thereby increasing excitability of hippocampal pyramidal neurons (Good and Murphy, 1996; Chen, 2005). Elevated Aβ also causes GABAergic dysfunction and attenuates excitatory synaptic transmission by decreasing the number of surface AMPA and NMDA receptors (Kamenetz et al., 2003; Hsieh et al., 2006; Shankar et al., 2007), as well as disrupting the development of aberrant synchrony in neural networks, damaging cognitive functions. Subsequent studies proved that neuronal activity regulates Aβ production (Kamenetz et al., 2003; Cirrito et al., 2005). Blocking neuronal electrical activity with TTX, a sodium channel blocker, decreased the cleavage of APP by β-secretase (Kamenetz et al., 2003). Recent studies found that blocking the network hyperactivity with the anti-epileptic drug lamotrigine, a voltage-dependent sodium channel inhibitor, reversed synaptic disorder and cognitive dysfunction in APP transgenic mice (Bakker et al., 2012; Sanchez et al., 2012; Zhang et al., 2014). These results indicate that Aβ, or other AD-related factors, plays a significant role in regulating neuronal activity at specific types of neurons as well as in wider neuronal networks, and Aβ and sodium channels have a certain relationship.

Voltage-dependent sodium currents play a critical role in action potential depolarization and firing frequency in many types of neurons (Kim et al., 2005; Baroni et al., 2013). We therefore hypothesized that the increased excitability induced by Aβ_1−42_ could be due, at least in part, to an upregulation of the Nav current. As we expected, the peaks of the voltage-dependent sodium current were significantly increased after Aβ_1−42_ treatment. The increased voltage-dependent sodium current could decrease action potential threshold and increase the probability of action potential generation in response to synaptic excitation, thus increasing the excitability of the neuron. However, Aβ upregulated sodium currents without significantly altering the voltage-dependence of activation and inactivation. We found that Aβ increased the expression of Nav and Nav1.6 in cultured neurons, indicating that the number of Nav channels could be altered by Aβ_1−42_. These results provide a possible mechanism for the increased excitability of pyramidal neurons previously observed after Aβ treatment. Confirming the causal relationship between the aberrant excitatory activity induced by Aβ and cognitive decline in AD patients would be an important insight into the pathogenesis of AD and provide new therapeutic avenues.

## Author contributions

Conceived and designed the experiments: XW, SL. Performed the experiments: XW, XZ, and CJ. Analyzed the data: XW, XZ, ZX, and QM. Contributed reagents/materials/analysis tools: TZ, NL. Wrote the paper: XW, XZ.

### Conflict of interest statement

The authors declare that the research was conducted in the absence of any commercial or financial relationships that could be construed as a potential conflict of interest.

## Supplementary material

Supplementary Figure S1 **Biochemical characterization of Aβ_1−42_**. Eighty nanogram aged Aβ_1−42_ and Aβ_1−42_ monomer samples were analyzed by SDS-PAGE and detected with anti-6E10 antibody. After 48 h aggregated Aβ_1−42_ with different molecular weight forms were displayed. aged presents Aβ_1−42_ with aggregation treatment; mono presents Aβ_1−42_ monomer.

Supplementary Figure S2 **Cell viability measurement of neuron treated with Aβ_1−42_**. Cell viability was determined by MTT assay in primary cultured neurons treated with 5 μM Aβ_42−1_ or 5 μM Aβ_1−42_ for 24 h. Aβ_1−42_ induced slightly loss of cell viability in neurons. (*n* = 3, means 3 independent experiments). Mean ± SEM was displayed. MTT, 3-(4,5-dimethylthiazol-2-yl)-2, 5-diphenyltetrazolium bromide. ^*^Presents *p* < 0.05 vs. control group.
