# Supplementary figures and images for: Elevated Neuronal Excitability Due to Modulation of the Voltage-Gated Sodium Channel Nav1.6 by Aβ1−42
Source: Front Neurosci. 2016 Mar 9;10:94. doi: 10.3389/fnins.2016.00094 (PMC4783403; doi:10.3389/fnins.2016.00094)

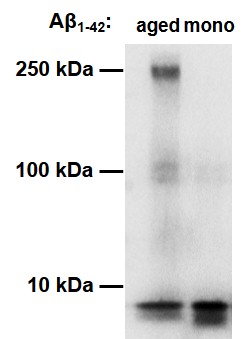

Supplement: Supplementary Figure S1 — Biochemical characterization of Aβ1−42. Eighty nanogram aged Aβ1−42 and Aβ1−42 monomer samples were analyzed by SDS-PAGE and detected with anti-6E10 antibody. After 48 h aggregated Aβ1−42 with different molecular weight forms were displayed. aged presents Aβ1−42 with aggregation treatment; mono presents Aβ1−42 monomer. [file Image1.JPEG]

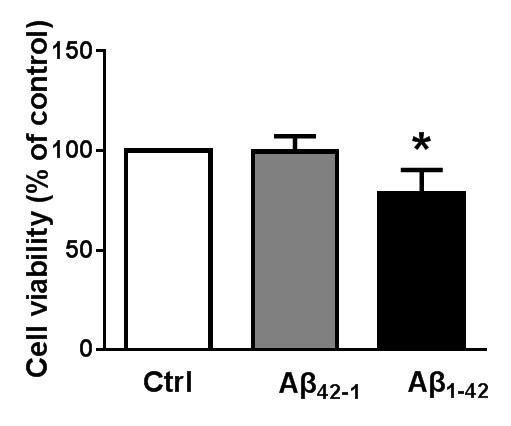

Supplement: Supplementary Figure S2 — Cell viability measurement of neuron treated with Aβ1−42. Cell viability was determined by MTT assay in primary cultured neurons treated with 5 μM Aβ42−1 or 5 μM Aβ1−42 for 24 h. Aβ1−42 induced slightly loss of cell viability in neurons. (n = 3, means 3 independent experiments). Mean ± SEM was displayed. MTT, 3-(4,5-dimethylthiazol-2-yl)-2, 5-diphenyltetrazolium bromide. *Presents p < 0.05 vs. control group. [file Image2.JPEG]
